# Clinical Efficacy of Trigeminal Nerve Stimulation in Pediatric ADHD: A Double-Blind Randomized Controlled Trial

**DOI:** 10.21203/rs.3.rs-11461111/v1

**Published:** 2026-10-30

**Authors:** Sandra K. Loo, Andrea Dillon, Giulia Salgari, Kelsey Stiles, Jennifer Cowen, Fang Yu Chang, Fiona Whelan, Catherine Sugar, James J. McGough

**Affiliations:** 1 Department of Psychiatry and Biobehavioral Sciences, Semel Institute of Neuroscience and Human Behavior, UCLA David Geffen School of Medicine, Los Angeles, CA, USA

## Abstract

Trigeminal Nerve Stimulation (TNS) represents a promising non-pharmacological monotherapy for pediatric Attention-Deficit/Hyperactivity Disorder (ADHD), yet its efficacy and underlying neurobiological mechanisms remain intensely debated. Here, we present a randomized controlled trial (N = 218) evaluating active (120 Hz) versus active-control (2 Hz) TNS in ADHD. Although robust active-control responses attenuated differences on traditional continuous symptom scales, blinded clinician-rated Clinical Global Impression of Improvement scales revealed a 35% binary response rate in the active cohort compared to 15% in the control arm (OR = 3.07; 95% CI: 1.45–6.24; *p* = 0.002). Concurrent quantitative electroencephalography demonstrated that active TNS selectively modulates right frontal alpha power—an effect moderated by biological sex, emerging in boys but not in girls. These data validate the clinical efficacy of TNS, highlight the semi-active nature of low-frequency cranial nerve stimulation, and provide a sex-specific neurophysiological framework for targeted pediatric neuromodulation.

## Introduction

Attention-Deficit/Hyperactivity Disorder (ADHD) is a highly prevalent neurodevelopmental disorder affecting an estimated 3% of the global population^1,2^, including millions of children and adults in the United States alone. While psychostimulants remain the clinical gold standard^3^, their therapeutic utility is severely constrained by suboptimal patient response, adverse side effects, and systemic barriers to healthcare access—leaving a massive clinical cohort critically undertreated. Extant non-pharmacological interventions, such as behavioral therapies, primarily mitigate secondary functional impairments rather than normalizing the core neurobiological deficits of inattention and hyperactivity^4^. Consequently, there is an urgent translational need for non-invasive, device-based neuromodulation therapies capable of directly targeting the aberrant neural circuitry underlying ADHD pathophysiology.

Trigeminal Nerve Stimulation (TNS)—a minimal-risk neuromodulation platform administered transcutaneously during sleep—represents a critical paradigm shift as the first device-based monotherapy cleared by the FDA for pediatric ADHD. In an initial randomized, double-blind trial, compared to sham control, active TNS significantly ameliorated clinician-rated ADHD symptoms and global functioning, yielding therapeutic effect sizes comparable to non-stimulant medications without inducing serious adverse events^5^. Mechanistically, TNS is posited to modulate top-down executive networks and attentional networks. Neuroimaging and quantitative electroencephalography (EEG) in clinical populations indicate that TNS induces focal metabolic alterations and broadband spectral power shifts within the anterior cingulate and prefrontal cortices—regions central to ADHD etiology^6–9^. These neurophysiological shifts are strongly coupled with downstream activation of ascending reticular activating structures, specifically the noradrenergic locus coeruleus and serotonergic raphe nuclei, effectively recalibrating the network-level excitatory-inhibitory balance required for sustained attentional control^10^.

Despite this therapeutic promise, the translational trajectory of TNS remains contentious. A recent randomized controlled trial failed to replicate these efficacy endpoints, reporting statistically indistinguishable differences in improvement on core symptoms between active TNS and a 2-Hz active control condition^11^. This discrepancy has introduced a critical controversy in the field, although it remains confounded by substantial methodological heterogeneities. Specifically, since the initial trial, some designs have introduced active low-frequency stimulation (2 Hz) rather than a true inert sham (0 Hz), expanded participant age ranges into adolescence, included lower-functioning cohorts (IQ ≥70), and permitted concomitant stimulant medication use in up to 39% of the sample^11^. Whether the observed lack of efficacy stems from an active therapeutic mechanism inherent to the 2-Hz control condition, or from sample and outcome-measure heterogeneity, remains unresolved.

Here, we present the largest efficacy trial of TNS in pediatric ADHD to date, utilizing an ethnically diverse cohort (*N* = 218). While our primary clinician-rated symptom endpoints echo the statistically overlapping trajectories observed in the Conti^11^ trial under the 2-Hz low-stimulation control paradigm mandated by the sponsor, we leverage multi-informant global functioning outcomes and objective neurophysiological metrics to reveal a distinct therapeutic response profile. Going forward, low stimulation (2 Hz) will be referred to as a *control* condition and no-stimulation (0 Hz) as a *sham* condition to differentiate these two conditions. By employing a multi-dimensional Clinical Global Impression of Improvement (CGI-I) framework, tailored to capture comprehensive adaptive functioning changes across home and academic environments, we unmask a stark divergence in treatment efficacy, demonstrating a 35% responder rate in the active TNS cohort compared to 15% in the control group. Furthermore, longitudinal EEG data confirm our core mechanistic hypothesis, demonstrating that positive therapeutic responses are tightly coupled with localized modifications in mid and right frontal spectral power^7^. By pairing granular TNS stimulation metrics with robust neurophysiological biomarkers, this study addresses existing clinical contradictions, establishes the biological potency of low-frequency cranial nerve stimulation, and refines the target responder population for device-based neurodevelopmental therapeutics.

## Results

### Participant Enrollment, Demographics, and Trial Disposition

A total of 296 individuals underwent screening, from which 219 eligible participants were randomized to receive either (120-Hz) active stimulation (n = 146) or low-stimulation (2-Hz) control (n = 73) Trigeminal Nerve Stimulation. In the active TNS cohort, one participant was withdrawn by investigators at baseline and seven participants withdrew prior to study completion, resulting in a completion rate of 97% (n = 138 active, n = 73 control; Fig. 1). Participants randomized to control were allowed to cross-over into open label active TNS treatment for four weeks; the open label data will be presented in the future. No statistically significant differences were observed in baseline demographic and clinical characteristics, which were balanced across both randomized arms (Table 1).

### TNS Adherence, Device Compliance, and Stimulation Parameters

Overall compliance to the protocol—defined as device usage ≥ 70% of nights during the four weeks was high at 92%. The active 120-Hz and 2-Hz control groups did not differ significantly with respect to parent-reported device use operationalized as total number of days worn, total hours worn per week, or mean duration of device usage on nights on which the device was worn (all *p’*s > 0.2). Importantly, the mean stimulation intensity was significantly higher in the control group (M = 3.95 mA, SD = 1.67; range 1.4–9.8 mA) than in the active group (M = 2.20 mA, SD = 0.90; range 0.69–5.74 mA; *F*_1,204_ = 97.8), *p* < 0.001; Extended Data Table 1).

### Primary and Secondary Efficacy Endpoints

General linear mixed models (GLMMs) were used to test for differential longitudinal trajectories in core ADHD symptomatology across the 4-week trial. We used a significance cutoff of α =.05 for the single primary symptom measure, .017 for the two secondary symptom measures (three total symptom outcomes). All other analyses were designated as exploratory and an alpha level of α =.01 was used to balance Type I and Type II error. For the primary, blinded clinician-rated ADHD-RS^12^ total score, the treatment group × time interaction was not statistically significant (*F*_4,845.82_ = 0.98, *p* = 0.42; Cohen’s *d*=.004), indicating that symptom reduction trajectories did not significantly diverge between the active (26% mean reduction) and control (22% mean reduction) arms. The lack of significant group separation was also observed across the individual inattentive and hyperactive-impulsive symptom dimensions that make up the ADHD Total score. This pattern was replicated on parent-reported SNAP^13^ total score, for which there was again no significant group × time interaction (*F*_4,835.86_ = 0.93, *p* = 0.45; Cohen’s *d*=.01). See Extended Data Table 2 for ADHD syndrome scale scores and statistics.

In contrast to single-dimensional symptom checklists, the secondary endpoint of blinded clinician-rated Clinical Global Impression of Improvement^14^ (CGI-I) at week 4—revealed a highly significant treatment divergence. CGI-I responders (i.e., scores of 1 [very much improved] or 2 [much improved]), who demonstrated holistic improvements in adaptive functioning across multiple settings, comprised 35% of the active 120-Hz cohort compared to only 15% of the control group (Fig. 2b). Logistic regression confirmed that children assigned to active TNS had a threefold higher probability of global clinical response relative to control (Odds Ratio [OR] = 3.07); 95% CI: 1.45–6.24; *p* = 0.002).

To further characterize the treatment responders, we fit GLMMs to symptom, functional and behavioral measures with treatment group, responder status and time, along with their interactions, as the predictors. For the ADHDRS, the three-way interaction was not significant (*F*_4,837.52_ = .82, *p* =.517), however, CGI-I responders exhibited ~49% average reduction in symptoms, whereas non-responders exhibited a nominal ~15% average reduction (*F*_4,837.52_ = 43.13, *p* <.001; Cohen’s *d*= .61 Fig. 2c). Similarly, improvement on the SNAP Total score among responders was, on average 41% while non-responders exhibited ~14% reduction in symptoms (*F*_4,837.52_ = 25.84, *p* <.001; Cohen’s *d*=.40). Concomitant improvements were also noted on the Weiss Functional Impairment Rating Scale^15^ (WFIRS), a validated measure of adaptive functioning, where again the three-way interaction including treatment group was non-significant (*F*_1,198_ =2.90, *p* =.09), but the responders showed significant end of trial improvement relative to non-responders (*F*_1,198_ =17.46, *p* <.001; Cohen’s *d*=.09; Fig 2d). The global clinical response in both treatment arms was further coupled with significant improvements across measures of executive control (BRIEF-2^16^ Global Executive Composite, *F*_1,205_ =16.60, *p* <.001; Cohen’s *d*=.15), and emotional dysregulation (ARIP^17^, *F*_1,193_ =16.58, *p* <.001; Cohen’s *d*=.09), although sleep architecture did not appear to be significantly altered (CSHQ^18^ Total Sleep Disturbance, *F*_1,205_ =1.20, *p* =.275; Cohen’s *d*=.09). Similarly, in an exploration of the 61% sub-sample with available teacher-rated ADHD symptoms (Conners Teacher Rating Scale^19^), the estimated effects were in the direction of improved ADHD behaviors for CGI-I responders (time x responder interaction: CTRS Inattention/Executive Dysfunction: *F*_1,128_ = 3.10, *p* =.08; Cohen’s *d*=.03; Hyperactivity: *F*_1,128_ = 3.98, *p*=.048; Cohen’s *d*=.03; Impulsivity: *F*_1,128_ = 3.28, *p* =.07; Cohen’s *d*=.03). See Extended Data Table 3 for additional information. This consistent pattern across a broad range of measures and informants shows that the CGI-I is capturing more than symptom recovery and reinforces confidence the finding of efficacy on that global scale.

### Neurophysiological Biomarkers and Longitudinal EEG Alterations

Resting-state EEG profiles were analyzed in 196 protocol-compliant treatment completers to evaluate the a priori neural co-primary outcomes, treatment related change in the EEG biomarker: right frontal (electrode F4) theta- and alpha-band power. We used a significance cutoff of α =.025 for the co-primary EEG outcomes and α = .01 for exploratory EEG analyses to balance Type I and Type II error. A significant treatment group × time interaction emerged selectively in the GLMM for right frontal alpha spectral power (F4; 8–12 Hz; *F*_1,194_ = 5.7, *p* = 0.018; Fig. 3a). The active TNS group exhibited a post-treatment attenuation of F4 alpha power, whereas the control cohort demonstrated stable or slightly elevated power trajectories. No significant group × time interaction was detected for right frontal theta power (F4; 4–7 Hz; *F*_1,194_ < .94, *p* = .333). However, treatment changes in right frontal theta power were tested as predictors of response, as reported in our previous paper^7^. A responder x time interaction trended toward significant for right frontal theta power (F4; 4-7 Hz, *F*_1,194_ = 4.65, *p* = 0.032; Fig. 3b) but not for alpha power (F4; 8-12 Hz, *F*_1,194_ = 1.59, *p* = 0.208). Similar responder by time effects were detected in frontal midline theta and alpha power (Fz; 4-7 Hz, *F*_1,194_ = 5.04, *p* = 0.026; Fz, 8-12 Hz, *F*_1,194_ = 4.65, *p* = 0.032), however, these effects do not survive correction for multiple comparisons. TNS responders exhibited decreased theta power at the end of treatment relative to baseline while nonresponders showed no power change at week 4. Overall, the location of TNS effects was confirmed in the right frontal region: alpha power change reflected treatment condition and theta power change was sensitive to treatment response.

### Biological Sex Stratified Neurophysiological Biomarker

Given that the observed reduction in alpha spectral power was inverted relative to pilot trial results^5^, and noting previously reported sexual dimorphism in endogenous brain oscillations^20,21^, we executed a sex-stratified mixed model analysis. This model unveiled specific effects driven by biological sex assigned at birth localized to the right frontocentral scalp. The corresponding 3-way interactions for tests of moderation by sex were not significant, although we were substantially underpowered due to the overall low response rate and the smaller size of the female cohort.

- **Male Cohort:** Males assigned to active TNS exhibited a significant reduction in right frontal alpha spectral power from baseline to week 4 relative to controls (electrode F4, 8-12 Hz, group by time interaction: *F*_1,141_ = 7.99, *p* = 0.005; Fig. 3a). A similar effect occurred for responder status in right frontal theta power (F4, 4-7 Hz, group by time interaction: *F*_1,141_ = 5.87, *p* = 0.017) Crucially, longitudinal alterations in right frontal theta and alpha power were highly predictive of clinical symptom reduction on the primary clinician-rated ADHD-RS Total scale (F4, 8-12 Hz, β = 1.20, t = 2.80, *p* = 0.005, 95% CI: 0.35–1.97; F4, 4-7 Hz: β = 1.10, t = 2.501, *p* = 0.013, 95% CI: 0.23-1.97).
- **Female Cohort:** Females demonstrated an entirely decoupled neurobehavioral profile with no significant change in either the active or control conditions in theta or alpha power according to response status (F4, 8-12 Hz, group × time interaction: *F*_1,51_ = .24, *p* = 0.63; F4, 4-7 Hz, group × time interaction: *F*_1,51_ = 0.44, *p* = 0.71). Moreover, these electrophysiological metrics failed to predict behavioral symptom changes (F4, 8-12 Hz: β = 0.03, t = 0.08, *p* = 0.94; F4, 4-7 Hz: β = 0.142, t = 0.37, *p* = 0.713; Fig. 3).

Despite the sex-specific EEG power modulations and differential associations with ADHD symptomatology, there were no significant sex differences in treatment-related change on ADHD symptom scales (see Extended Data Tables 5 & 6).

### Biophysical Dosing Dynamics: Active versus Semi-Active Stimulation Profiles

To test the hypothesis that the 2-Hz control paradigm acted as a biologically active central stimulus, we ran a comprehensive correlation matrix mapping TNS stimulation parameters against longitudinal EEG spectral power shifts. Within the 2-Hz control arm, clear linear biophysical relationships were established: higher average stimulation intensity was strongly correlated with theta and alpha power modulations across midline and right-hemisphere sensors, while the mean duration of nocturnal device usage (hours per week) significantly correlated with midline frontal theta and beta power shifts (Table 2). Conversely, within the active 120-Hz cohort, these parametric correlations were entirely absent, with the exception of two isolated trend-level associations.

Fisher’s *r*-to-*z* transformations were executed to formally compare the strength of these correlation coefficients between the randomized arms, confirming that the low-stimulation control condition maintained significantly stronger associations with localized neurophysiological modifications than the active condition. Specifically, at right frontal electrode F4, the positive correlation of r = 0.34 between theta power changes and average stimulation level in the control group was significantly stronger than the negligible association of r = −0.02 in the active group (difference in correlation *z* = 2.4, *p* = 0.02). This pattern was replicated at adjacent electrode FC4, where theta power (4–7 Hz) modifications displayed a significantly robust coupling with control stimulation intensity of *r* = 0.43 relative to active parameters where the correlation was *r* = 0.008 (difference *z* = 2.9, *p* = 0.004). Furthermore, the mean number of hours of stimulation per week—was more strongly associated with midline frontal (Fz) theta power shifts in the control cohort *r* = 0.418, than in the active cohort *r* = 0.04 (*z* = 2.6, *p* = 0.002). Within the 2-Hz control environment, escalating stimulation intensity and extended duration of exposure were systematically tied to localized EEG power changes, confirming a linear dosing dynamic that contrasts with the threshold-driven saturation profiles observed under active 120-Hz exposure.

### Adverse Events, Side-Effect Profiles, and Tolerability

No serious adverse events (AEs) occurred during the trial, and no participants withdrew due to treatment-emergent medical complications. Weekly clinician-solicited evaluations indicated that all recorded AEs were mild to moderate in severity, and the incidence of treatment-related AEs did not significantly differ between the active and control arms (all *p*’s > 0.2; see Extended Data Tables 7, 8). Safety monitoring via the Children’s Depression Inventory^22^ (CDI-2) showed that the cumulative frequency of endorsed suicidal ideation, if anything, progressively declined across the 4-week trial period (13% at baseline down to 11% at week 4). Secondary clinical diagnostic reviews of all endorsed ideation confirmed that no participant presented active clinical risk or required acute intervention. Overall, transcutaneous TNS demonstrated excellent tolerability with a benign safety signature.

### Assessment of Trial Blinding Fidelity

The integrity of the double-blind architecture was systematically verified at week 1 and week 4. At week 1, parental treatment expectations and initial perceptions of symptom mitigation evaluated via the Early Impressions Questionnaire^23^ demonstrated no significant differences between the two arms (all *p*’s > 0.16). At week 4, parents were required to guess their child’s treatment allocation. Indecision was evenly distributed across cohorts, with “don’t know” endorsed by 41% of parents in the active group and 40% in the control group. Among parents who attempted a guess, 38% of the control arm and 26% of the active arm correctly identified their group assignment. A chi-square analysis confirmed that allocation guess distributions did not significantly diverge between the cohorts (χ^2^ = 0.61, *p* = 0.74), demonstrating that successful treatment blinding was preserved throughout the execution of the trial.

## Discussion

This study represents the largest double-blind, randomized controlled trial of external trigeminal nerve stimulation for pediatric ADHD to date, offering key clinical and neurophysiological insights that reshape our understanding of non-pharmacological neuromodulation. On traditional, multi-informant ADHD symptom rating scales, both the high-stimulation active (120 Hz) and low-stimulation control (2 Hz) cohorts exhibited a substantial 20–25% average reduction, with no divergence between the groups. However, evaluating efficacy through traditional symptom checklist dimensions alone obscures a significant therapeutic effect.

By utilizing blinded clinician ratings via the Clinical Global Impression of Improvement (CGI-I) scale—which integrates broader functional domains including adaptive functioning, executive functioning, emotional dysregulation, and interpersonal relationships—we unmasked a stark divergence in treatment efficacy. Active TNS yielded a 35% clinical responder rate compared to just 15% in the control cohort. This threefold increase in odds of response probability is tightly coupled with significant secondary improvements across validated metrics of daily function (WFIRS), executive control (BRIEF), and emotional lability (ARIP). Crucially, a ~50% reduction in symptom severity on average separated global clinical responders from non-responders, aligning with established benchmarks in major neuropsychiatric trials^24,25^.

A primary challenge in interpreting recent pediatric TNS literature—specifically the overlapping trajectories noted by Conti and colleagues^11^ —is the reliance on a low-frequency (2 Hz) control condition mandated by the funding agency. Studies in adult treatment resistant depression report behavioral improvements in depressive symptomatology and neurophysiological power modulation with low frequency TNS stimulation (2 Hz & 20 Hz^26^; 10 Hz^27^). Our cross-trial synthesis indicates that while the inert, no-stimulation sham (0 Hz) utilized in our earlier study^5^ failed to drive meaningful behavioral changes, the 2-Hz control condition behaves not as an inert placebo, but as a semi-active therapeutic intervention capable of inducing ADHD symptomatic improvement but falling short of achieving similar rates clinically significant improvement in global, macro-level adaptive functioning (see Figure 4).

This semi-active classification is strongly substantiated by our quantitative EEG data. Within the 2-Hz control group, both higher stimulation intensity and longer duration of device usage significantly correlated with localized theta and beta power modulations at midline and right frontal regions. Although the 2-Hz paradigm delivered a lower density of pulses per second, the cumulative nocturnal dose remained biologically meaningful. Over a standard 10-hour sleep window, a child in the control group received approximately 600 pulses per night, culminating in an aggregate exposure of roughly 16,800 pulses across the 28-day trial. Positron emission tomography (PET) mapping in treatment-resistant depression demonstrates that an equivalent acute electrical dose (14,400 pulses) is sufficient to induce immediate metabolic activation within the right medial frontal gyrus, left Inferior frontal gyrus, and bilateral anterior cingulate cortex^6^—the precise hub networks implicated in top-down attentional control and ADHD pathophysiology^28^. Beyond pure pulse count and electrical field amplitude, the high stimulation intensity tolerated by the control group (mean = 4.0 mA) interacted synergistically with low-frequency delivery to generate an electrical energy density capable of depolarizing ophthalmic *V*_1_ branch fibers of the trigeminal nerve^29^. Emerging neurophysiological data show that brief, low-frequency TNS bursts reliably trigger downstream locus coeruleus activation, marked by increased pupil dilation and suppressed P300 amplitudes^30,31^, which serve as established proxies for central noradrenergic and serotonergic transmission.

Intriguingly, while the 2-Hz control cohort displayed linear correlations between stimulation parameters and EEG power shifts, the 120-Hz active group exhibited virtually no such parameter-dependent correlations. This discrepancy strongly points toward a circuit-level threshold or saturation effect. At higher frequencies (120 Hz), the targeted frontoparietal networks likely achieve maximum neuroplastic engagement or neurotransmitter saturation, rendering individual variations in current intensity or nightly compliance secondary to the dominant frequency effect. Taken together, these data clarify the discrepancies in the field: low-frequency TNS provides a sub-maximal yet biologically active stimulus that modifies underlying frontoparietal circuitry, whereas high-frequency TNS drives an optimal, systemic reorganization of global adaptive functioning. Future biophysical modeling must systematically dissect the interlocking variables of frequency, intensity, and developmental neural plasticity to fully map the therapeutic boundaries of device-based neurodevelopmental interventions.

An unexpected yet significant finding of this trial was the apparent differential impact of sex assigned at birth on TNS neurophysiological effects. Although the hypothesized right frontal (F4) spectral power biomarker demonstrated a significant treatment condition by time interaction effect, the direction of this power shift was inverted relative to prior pilot data. In our previous study, there was a higher percentage of girls in the sample, thus shifting mean group changes in the opposite direction. This electrophysiological divergence strongly implies an underlying sexual dimorphism in endogenous brain theta and alpha-band dynamics^21,32–35^ and trigeminal nerve architecture^36^. Morphological and clinical literature indicates marked sex-based variations in trigeminal system pathophysiology; for instance, idiopathic trigeminal neuralgia exhibits a significantly higher prevalence in females^37^, potentially driven by anatomical variations such as a constricted posterior fossa^38^, distinct genetic vulnerabilities^39^, and fluctuating hormonal profiles^40^. We hypothesize that these dimorphic structural and neurochemical traits may alter the mechanical or electrical activation threshold in female participants^37^, rendering them highly sensitive to even the nominal energy delivered by the 2-Hz control paradigm. The small sample size of female participants in this trial constrains fully powered analysis of the sex moderation effect. Dedicated, sex-powered cohorts are critically required to fully untangle these dimorphic neurodevelopmental responses to cranial nerve stimulation.

While this trial features a rigorous double-blind architecture and a large, ethnically diverse cohort, several methodological limitations must be considered. First, the a priori primary and secondary symptom measures (ADHD-RS and SNAP) did not statistically diverge between the active and control arms. Because the trial’s initial power calculations were designed to detect a contrast against an inert, no-stimulation sham (0 Hz) rather than an active-control (2 Hz) paradigm, the analytical model was underpowered (post-hoc power = 34%) to detect symptom-scale separation despite the large overall sample size. Similarly, while the F4 electrophysiological biomarker achieved statistical significance, the sex-stratified inversion of the alpha power direction indicates that pooling heterogeneous cohorts can mask distinct biophysical phenotypes. Consequently, our sub-analyses remain post-hoc and require independent replication within a formal multi-arm, dose-ranging framework (e.g., 0 Hz, 2 Hz, 20 Hz, and 120 Hz) and stratification of randomization by sex with a larger female cohort.

Second, the multi-informant paradigm was constrained by a high rate of missing teacher-rated data, limiting our longitudinal behavioral matrix. Relying on parent-reported behavior and compliance introduces inherent recall bias and systematic error variance. This challenge was compounded by technical unreliability in the device’s hardware compliance timer, which failed to correlate with subjective logs, thereby precluding a completely objective verification of exact nocturnal electrical dosing. Finally, a baseline placebo response must be acknowledged; notably, the 15% CGI-I responder rate observed in our 2-Hz control group perfectly mirrors the 15% clinical response rate elicited by the completely inert 0-Hz sham in our previous pilot trial, suggesting that while the 2-Hz control alters neurophysiology, a subset of behavioral improvement remains driven by expectancy and trial-adherence effects.

In conclusion, this large-scale, double-blind randomized controlled trial demonstrates that high-frequency (120 Hz) trigeminal nerve stimulation drives a clinically significant, comprehensive improvement in global adaptive functioning—encompassing core ADHD symptoms, executive dysfunction, and emotional dysregulation—in 35% of children receiving active TNS compared to 15% in the low-frequency control group. Although the active and control arms exhibited overlapping trajectories on isolated symptom checklists, responder analyses demonstrate that meaningful global clinical recovery is associated with ~50% symptom reduction coupled with multidimensional functional improvements. From a translational perspective, optimizing real-world global adaptive capacity represents a highly valuable therapeutic target for neurodevelopmental disorders. Given the high rate of family compliance, successful treatment fidelity, and an exceptionally benign side-effect profile, transcutaneous TNS represents a safe, mechanistically viable, and clinically meaningful non-pharmacological monotherapy for pediatric ADHD.

## Online Methods

### Study Design and Ethics

This study was a 4-week randomized, double-blind, active-controlled confirmatory trial evaluating the efficacy and safety of trigeminal nerve stimulation (TNS) in pediatric attention-deficit/hyperactivity disorder (ADHD). Participants who met eligibility criteria were randomized 2:1 to active vs. control TNS and used the device nightly for 4 weeks (Phase 1). The double-blind trial was followed by Phase 2, a subsequent 4-week, open-label, single-arm extension phase for participants randomized to the control arm during Phase 1. Phase 2 was included primarily out of ethical responsibility to provide study subjects initially randomized to control with active treatment. Data from the Phase 2 open-label extension will be reported separately. The study enrollment period started August 2022 and ended February 2026; follow up assessments continued until September 2026.

This study was originally proposed as a multisite study with an additional data collection site. Due to regulatory concerns, the second site was suspended by the study sponsor and not allowed to restart study activities; this study was carried out as a single site study with permission from sponsor. Consequently, all study procedures and assessments were conducted in offices and laboratories at UCLA. This study is registered at clinicaltrials.gov under NCT05374187; the study protocol and statistical analysis plan can be accessed from this site.

All procedures were approved by the UCLA Medical Institutional Review Board 3 and conducted in strict accordance with the Declaration of Helsinki. Prior to the initiation of any screening procedures, parents or legal guardians provided written informed permission, and child participants provided written assent.

### Participant Recruitment and Eligibility

Participants were recruited via community advertisements, online postings, and direct clinical referrals. Eligible participants were children aged 7–12 years with a primary diagnosis of ADHD (any current presentation) established via the Schedule for Affective Disorders and Schizophrenia for School-Age Children^41^ (**KSADS)** semi-structured diagnostic interview and a corroborating clinical evaluation.

Inclusion criteria were:

- A minimum score of 24 on the clinician-administered, parent-reported ADHD Rating Scale-5^12^ (**ADHD-RS-5**).
- A baseline Clinical Global Impression-Severity^14^ (**CGI-S**) score ≥4 (indicating moderate to severe impairment).
- No current medication with CNS effects. Participants previously on psychostimulant medication were required to be not optimally treated and off medication for one week or 5 half-lives for all other medications.
- An estimated full-scale intelligence quotient (**FSIQ**) ≥ 80, determined using two subtests of the Wechsler Abbreviated Scale of Intelligence^42^ (**WASI**).
- Parent and participant able to complete rating scales and other measures in English.
- Participant able to cooperate during EEG.

Exclusion criteria were:

- Impaired functioning to a degree that required immediate initiation of ADHD medication in the opinion of the parents and/or investigator.
- Current diagnosis of autism spectrum disorder or major depression.
- History of lifetime psychosis, mania, or seizure disorder.
- Active suicidal ideation at baseline.
- History of seizure disorder or head injury with loss of consciousness.

All enrolled children were medication-free and remained completely free of concurrent ADHD medications throughout the trial duration.

### Randomization and Masking

Participants who met eligibility criteria at screening participated in baseline assessments and were randomized in a 2:1 ratio to receive either active or control TNS. Randomization was stratified on the Working Memory t-score (≥70 vs. <70) of the parent-completed Behavior Rating Inventory of Executive Function, Second Edition^16^ (BRIEF-2). Allocation sequences were generated by the institutional biostatistics team via a web-based randomization tool using a random mixture of block lengths of 3 and 6 to preserve concealment. The single, unblinded research associate received the participant’s treatment allocation, selected the appropriate device (active or control) and demonstrated its use to parent and child together during the baseline visit; the same device was taken home by the participants and utilized for the four-week trial.

To maintain strict double-blind conditions, active and control TNS systems were identical in external appearance, acoustic output, user interface, and operational feedback. Participants, their families, and all clinical and psychometric investigators remained blinded to the treatment allocation during Phase 1. Complete unblinding occurred only after the completion of all Week 4 assessments. A single designated unblinded research assistant who had no involvement in clinical or behavioral assessments provided TNS instruction to families, assisted with device trouble shooting, and managed device inventory, programming, and allocation logs.

### Trigeminal Nerve Stimulation Protocol

Stimulation was delivered using the Monarch TNS System (NeuroSigma, Inc.). The stimulation device was secured to the participant’s sleepwear and connected via thin, insulated lead wires to disposable, bilateral, silver-gel, self-adhesive patch electrodes. Parents applied the patches across the child’s forehead nightly to achieve transcutaneous, bilateral stimulation of the ophthalmic branch (*V_1_*) of the trigeminal nerves.

Participants were instructed to wear the device for approximately 8 hours per night during sleep throughout each 4-week intervention period. Initial stimulation thresholds were determined via a standard titration protocol during the baseline clinic visit. Thereafter, parents adjusted the current amplitude nightly in 0.2 mA increments within a predefined safe range (0–10 mA) to identify a stimulation level immediately below the child’s subjective threshold of sensory discomfort. Electrodes were removed and discarded each morning upon waking.

The experimental conditions were parameterized as follows:

- **Active TNS:** Delivered a continuous 120-Hz repetition frequency with a 250-μs pulse width. The stimulation operated on a duty cycle of 30 seconds on, 30 seconds off.
- **Low-frequency Control TNS:** Formulated to enhance blinding fidelity compared to completely inert sham systems. The device delivered electrical stimulation at a 2-Hz repetition frequency and a 50-μs pulse width for 30 seconds at the beginning of each hour. During the remaining hour, the current was automatically shunted through an internal resistor inside the device enclosure to mimic the battery drain profile and hardware warmth of the active unit without delivering peripheral nerve stimulation.

### Assessment Schedule and Blinding Validation

After the baseline assessment, clinician- and parent-completed symptom and functional outcome ratings, targeted adverse event solicitations, and a standardized side-effects rating scale were completed weekly. At Week 1, parents completed the Early Impressions Questionnaire^23^ to quantify baseline expectations and treatment credibility. At Week 4, parents completed a validated blinding questionnaire to guess their child’s treatment assignment and evaluate overall intervention satisfaction. Full clinical and neurophysiological assessment batteries—comprising teacher-completed behavioral ratings, computerized attention tasks, anthropometric metrics (height and weight), and resting-state EEG recordings—were obtained at baseline and at the end of the double blind trial (Week 4).

### Electroencephalographic Data Acquisition

Five minutes of continuous, resting-state, eyes-open EEG data were recorded at baseline and Week 4. Data were acquired using a 128-channel high-impedance Hydrocel Geodesic Sensor Net connected to a GES300 system (Electrical Geodesics, Inc.). Raw signals were referenced to the central vertex electrode (Cz), sampled at a rate of 1,000 Hz. Electrode impedances were maintained below 50 kOhms throughout the recording session in accordance with manufacturer recommendations. Ocular artifacts were monitored continuously via bipolar electrode configurations placed on the outer canthus of each eye to capture horizontal movements (HEOG) and superior/inferior to the orbits to capture vertical eye movements and blinks (VEOG).

### Electroencephalographic Preprocessing and Spectral Analysis

Continuous raw EEG records were exported and processed within the EEGLAB v. 2025.0.0^43^ environment running in MATLAB R2020a^44^. The preprocessing pipeline utilized the following sequential steps:

1. **Filtering:** Data were filtered with a 1Hz high-pass filter and a 55Hz low-pass filter and then corrected to remove significant non-stationary artifacts using Artifact Subspace Reconstruction^45^ (ASR) with the burst criterion setting as 8.
2. **Re-referencing:** Signals were computationally transformed from the acquisition reference (Cz) to a common average reference configuration.
3. **Independent Component Analysis (ICA):** Cleaned data matrices were decomposed using Adaptive Mixture Independent Component Analysis^46^ (AMICA) algorithm to isolate distinct neural and non-neural signal sources.
4. **Artifact Rejection:** ICLabel^47^ machine learning algorithm was utilized to generate the maximal prediction confidence for each independent component (IC) that isolated artifact sources (i.e., muscle, eye, heart, line noise, and channel noise) from the signal or had residual variance ≥20%. These ICs were then subtracted from the EEG data prior to analysis.
5. **Back projection to channel space**: The remaining physiological brain components were back-projected into channel space.
6. **Segmentation**: Following cleaning, the continuous recordings of resting state (EO) data were segmented into 2-second, non-overlapping epochs. Excessively noisy epochs were identified based on the statistical properties of the time series, using a 5-standard-deviation threshold. Epochs were rejected in a single pass with pop_autorej.m function in EEGLAB. The average within-individual EEG data retention rate was 95.9% for Baseline visit and 95.5% for Week 4.
7. Fast Fourier Transform (FFT) algorithms estimated absolute spectral power μV^2^ averaged across standard clinical frequency bands: Delta (1–3 Hz), Theta (4–7 Hz), Alpha (8–12 Hz), Beta (13–25 Hz), Gamma (30–40 Hz)

Spectral analyses focused specifically on a priori EEG biomarker outcomes, specifically right frontal (electrode F4) theta (4-7 Hz) and alpha (8-12 Hz) spectral power, based on our previous findings^7^. Theta and alpha power in other frontal and frontocentral electrodes (F3, Fz, FC4, FCz, FC3) were included as exploratory EEG outcomes and to provide contrast for specificity of effects. Frontal channels correspond with reported TNS stimulation effects in neuroimaging studies^6,8–9^. Other frequency bands were examined as neural correlates of symptom and functional measures along with TNS stimulation metrics.

### Outcome Metrics

- **Primary Efficacy Outcome:** The primary clinical outcome measure was the clinician-administered ADHD Rating Scale-5 (ADHD-RS-5) Total Score, synthesized from comprehensive parent interviews and all available clinical data. The contrast of interest was the differential change between the treatment arms over the 4-week active intervention period
- **Secondary Efficacy Outcomes:** Secondary outcomes included the clinician-rated Clinical Global Impression-Improvement^14^ (CGI-I) scale at Week 4 (the target endpoint) and the parent-completed Swanson, Nolan, and Pelham^13^ (SNAP) Rating Scale, for which the contrast was differential change over the 4-week intervention window. Clinician ratings for ADHD symptoms and CGI-I functional trajectories were calibrated using standardized behavioral anchors to ensure cross-rater reliability.
  The CGI-I score was not determined solely by changes in core ADHD symptoms; instead, it reflected a holistic clinical synthesis of the child’s functioning across home, academic, and peer environments. This assessment was corroborated by validated instruments measuring functional impairment (Weiss Functional Impairment Rating Scale^15^; WFIRS), executive functioning (the Behavior Rating Inventory of Executive Function, Second Edition^16^; BRIEF-2), and emotion dysregulation (the Affective Reactivity Index–Parent Version^17^; ARI-P). For categorical response analysis, participants achieving a CGI-I score of 1 (Very Much Improved) or 2 (Much Improved) were classified as treatment responders, whereas those scoring 3 (Minimally Improved) or 4 (No Change) were classified as non-responders.
- **Exploratory Outcomes:** Exploratory endpoints measured at baseline and Week 4 included the clinician-completed CGI-Severity^14^ (CGI-S) scale; parent- and teacher-completed forms of the Conners 4 Short Form^8^ (Conners-4); and parent-reported metrics from the BRIEF-2, Children’s Sleep Habits Questionnaire^18^ (CSHQ), the WFIRS, and the ARI-P. Self-report measures completed by the child participants at baseline, Week 4, and Week 8 included the Affective Reactivity Index^17^ (ARI-C), the Revised Child Anxiety and Depression Scale-25^48^ (RCADS-25), and the Children’s Depression Inventory-2^22^ (CDI-2). Exploratory outcomes not reported here will be presented separately in the future.
- **Neurocognitive and Physiological Assessments:**
  Resting state EEG was collected with eyes open for 5 minutes. Computerized cognitive tasks were administered concurrently with EEG recordings at baseline and at the conclusion of each study phase. These tasks comprised a standard Go/No-Go paradigm to quantify sustained attention motor response inhibition, and the Happy Café task to evaluate implicit emotional processing. Physiological monitoring included objective, standardized assessments of participant height and weight collected at baseline, Week 4, and Week 8. EEG data collected during resting state eyes closed and computerized cognitive tasks will be reported separately.
- **Compliance and Safety Monitoring:** Intervention adherence was rigorously tracked using a daily parent-completed sleep diary spanning the entire duration of the trial. For each night of treatment, parents documented device application and removal timestamps (device-on and device-off hours), alongside the specific stimulation current intensity (μA) utilized. These diary metrics were systematically collected and entered into the study database on a weekly basis during concurrent clinic visits, while parents completed the scheduled behavioral and side-effects ratings. An *a priori* adherence compliance threshold was established, requiring participants to successfully wear the device for ≥ 70% of nights over the 4 week double-blind trial. Safety and tolerability were monitored via weekly standardized adverse event inquiries and parent-completed Side Effects Rating Scales. Treatment-emergent suicidality was rigorously monitored using the self-report CDI-2 suicide ideation subscale items administered directly to the child. Clinical assessment followed any report of suicidal ideation and appropriate steps taken to ensure the child’s safety.

### Statistical Analysis Plan and Power

All analyses were conducted in SPSS v 29^49^. Prior to performing the primary efficacy assessments, we obtained graphical and descriptive statistics to characterize the sample, check for outliers or other distributional issues, and examine the forms of the longitudinal trajectories. Specifically, we used means (SDs) and proportions, along with t-tests and chi-squared/Fisher exact tests as appropriate, to compare the intervention arms on (a) baseline demographic and clinical characteristics (to verify the balancing effects of randomization), (b) adherence/device compliance, stimulation parameters, checks of blinding and other structural measures, and (c) adverse events/side effect profiles and treatment satisfaction scores.

Our primary efficacy aims involved comparing changes on clinical, behavioral, and neural measures between the active and control conditions across the double-blind trial from baseline to end of treatment (week 4) Our principle analytical technique was thus the Generalized Linear Mixed Model^50^ (GLMM). GLMMs provide a unified analytical framework as they include correlation and regression analyses as special cases, accommodate a broad range of outcome distributions and variable types via use of appropriate link functions, allow for the inclusion of both fixed and time-varying covariates, appropriately account for the correlations induced by repeated measurements within person, and produce unbiased parameter estimates as long as observations are missing at random^50,51^ (MAR). Primary models were fit on an intent-to-treat (ITT) basis, using all available measurements on all subjects, regardless of the degree of study participation, and were run without covariate adjustment following standard practices^52^. Follow-up models were used to explore the effects of demographic, clinical and structural measures. To protect against Type I errors, we used a significance cutoff of α =.05 for the efficacy test of the single primary symptom measure, .017 for the two secondary symptom measures (three total symptom outcomes) and .025 for the co-primary EEG outcomes. All other analyses were designated as exploratory and an alpha level of *p* < .01 was used to balance Type I and Type II error. Cohen’s *d* was calculated using the pooled baseline standard deviation of each efficacy measure.

To examine the effects of TNS on symptoms, we fit a GLMM with the primary ADHD-RS total score as the outcome. The predictors were treatment group (active or control) as the between-subjects factor, time (0, 1, 2, 3 and 4 weeks) as the within-subjects factor, a group by time interaction to test for differential treatment effects (our primary target), and subject-level random intercepts (equivalent to a compound symmetric covariance structure) to account for the correlations induced by repeated measurements within participant. Time was treated as a factor since recent data^11^ as well as our own graphical analyses suggested a non-linear pattern of change, with faster initial improvement followed by a gradual leveling off. The model was fit using restricted maximum likelihood estimation (REML) with Satterthwaite degrees of freedom. A parallel model was used to examine efficacy for the SNAP total score (secondary continuous outcome) and the inattentive and hyperactive-impulsive subscales for both the ADHD-RS and SNAP (presented in Extended Data). For the CGI-I (binary secondary outcome), our primary hypothesis of a difference in response rates at week 4, corresponded to a chi-squared test of independence but we embedded it in a logistic regression framework to allow for follow-up analyses including covariates. For the core EEG measures (theta and alpha power in right frontal (F4, primary) as well as exploratory outcomes (theta and alpha power in other frontal and frontocentral electrodes (F3, Fz, FC4, FCz, FC3) that were measured at baseline and week 4, we used the same GLMM approach but with a less detailed time trajectory.

Because we observed a significant difference in rates of clinical response at 4 weeks but did not find a differential pattern of change on the primary symptom measure, we performed three key types of follow-up analyses to help elucidate our findings. First, we compared the outcome trajectories for subjects designated as CGI-I treatment responders or non-responders on a range of symptom, behavioral, neural, and structural measures to try to pinpoint what was driving global clinical improvement beyond functioning. This was done using the same GLMM approach as for the primary aims. Second, we performed sex stratified analyses of the EEG measures due to the observed power inversion and previously reported sexual dimorphism in EEG theta and alpha power^20,21^. The resulting patterns are suggestive and consistent with previous findings. (The corresponding 3-way interactions for tests of moderation by sex were not significant, although we were substantially underpowered due to the overall low response rate and the smaller size of the female cohort.) Finally, we performed correlational analyses, examining the relationships of TNS usage levels and intensity parameters with the EEG outcomes, although this should not be interpreted as a formal dose-response assessment since stimulation levels and length of time worn were not randomized.

#### Power:

The original study design called for recruiting 280 participants to achieve a target of 225 completers (150 active and 75 control), allowing for 20% screen failure, attrition and data loss during the double-blind phase. These target calculations were based on the results from the initial pilot trial^5^ and assumed a two-sided significance level of α=.025, correcting for two pre-specified primary outcomes within each efficacy aim (ADHD-RS Total Score and CGI-I response for symptom outcomes; F4 theta and alpha spectral power for resting EEG); and, where applicable, assumed a moderate within-subject correlation of r=0.5. Under these assumptions, for the ADHD-RS, the GLMM with 5 timepoints had 80% power^53^ to detect a group by time interaction effect size of f^2^=.008, equivalent to a linear change from no difference in ADHD-RS symptoms at baseline to a difference of ~d=0.4. For the binary CGI-I outcome, the proposed design had 80% power to detect a difference of 24% or higher in response rates between the active and control TNS groups at 4 weeks. For the EEG models, with only two time points, the proposed sample size provided 80% power for an interaction effect size of f^2^=.013, equivalent to a change from no difference at baseline to ~d=0.5 at four weeks. We also note that for follow-up tests of association this proposed sample size provided 80% power to detect a correlation of r = 0.20 between change in EEG and change in symptoms, across the entire sample or r = 0.25 in the active TNS arm. To allow for the possibility of attenuated results in a validation trial with a non-sham control, these targets were conservative compared to the results reported in the pilot trial, which included a significant interaction with a group difference of d=.5 at end of treatment on the ADHD-RS, a difference of 38% difference in CGI-I response rates (52% in active TNS vs. 14% for a Sham control), differential changes of d = 0.9 and 0.6, respectively, in theta and alpha power, and a correlation of r = −0.3 between change in theta power and change in symptoms.

Prior to initiating the trial reported here, in consultation with the sponsors, the core efficacy targets were adjusted, to designate the ADHD-RS as the sole primary symptom outcome, with CGI-I response and the SNAP as secondary; the co-primary EEG measures remained unchanged. We screened 296 subjects, of whom n=219 were eligible and randomized (145 to active TNS and 73 to the low-frequency control condition); one participant was withdrawn at baseline due suicidality and only 7 of the randomized subjects, all in the active TNS arm, failed to complete the protocol, an acute drop-out rate of 3.2%, Thus, the obtained sample size was extremely close to the original target. However, the observed effect size for the primary symptom endpoint was only d = .23 (difference in change scores relative to baseline standard deviation), about half what was originally projected, and the groups did not appear to separate at all until week 4. Consequently, the post-hoc (achieved) power for the change from no difference at baseline to a difference at the end of acute treatment was only 34%.

## Extended Data

**Table ED1.** TNS Stimulation metrics by treatment condition

	Group	Mean	Std. Error	F (1,205)	P
Total Hours per Week	Control (n=70)	65.2902	0.70238	1.463	0.228
	Active (n=137)	63.901	0.73782		
Average Hours per night	Control	9.7072	0.09568	0.276	0.6
	Active	9.636	0.08361		
Avg_Stim_Intensity (mA)	Control	3.9487	0.19926	98.754	<.001
	Active	2.2026	0.07379		
TNS_Compliance (>70% for 3 weeks)	Control	0.9857	0.01429	6.064	0.015
	Active	0.8897	0.02696		

**Table ED2.** ADHD symptom scores by randomization group

	Active (n=138)		Control (n=73)					
	Mean	Std. Error	Mean	Std Error	aMD	95% CI	Cohen’s *d*	group x time p
**ADHDRS Total**								
Baseline	35.84	0.75	35.15	1.06				
Week 1	30.06	0.75	30.40	1.06	0.33	−2.22 - 2.88	0.02	
Week 2	28.61	0.75	28.03	1.06	0.58	−1.97 - 3.13	0.04	
Week 3	26.86	0.76	26.17	1.06	0.69	−1.87 - 3.24	0.04	
Week 4	26.72	0.76	27.58	1.06	0.85	−1.70 - 3.41	0.05	0.42
**ADHDRS Inattention**								
Baseline	19.55	0.39	20.25	0.55				
Week 1	16.42	0.39	17.81	0.55	1.39	0.06 - 2.72	0.17	
Week 2	15.62	0.39	16.32	0.55	0.70	−0.63 - 2.02	0.09	
Week 3	14.61	0.40	15.37	0.55	0.77	−0.57 - 2.10	0.09	
Week 4	14.71	0.40	15.99	0.55	1.27	−0.06 - 2.60	0.15	0.64
**ADHDRS Hyp/Imp**								
Baseline	16.30	0.52	14.90	0.74				
Week 1	13.65	0.53	12.59	0.74	1.06	−0.72 - 2.84	0.10	
Week 2	12.99	0.53	11.71	0.74	1.27	−0.51 - 3.05	0.12	
Week 3	12.26	0.53	10.79	0.74	1.47	−0.32 - 3.25	0.13	
Week 4	12.02	0.53	11.59	0.74	0.43	−1.36 - 2.21	0.04	0.45
**SNAP Total**								
Baseline	36.39	0.82	34.96	1.15				
Week 1	32.39	0.82	31.77	1.15	0 62	−2.16 - 3.40	0.04	
Week 2	29.34	0.82	29.15	1.15	0.19	−2.59 - 2.97	0.01	
Week 3	27.79	0.82	26.82	1.15	0.97	−1.82 - 3.76	0.06	
Week 4	27.94	0.82	28.51	1.15	0.57	−2.21 - 3.34	0.03	0.59
**SNAP Inattention**								
Baseline	20.32	0.44	20.60	0.62				
Week 1	17.84	0.45	18.69	0.63	0.85	−0.66 - 2.36	0.09	
Week 2	15.97	0.45	16.94	0.63	0.97	−0.54 - 2.48	0.10	
Week 3	15.29	0.45	15.70	0.63	0.41	−1.10 - 1.93	0.04	
Week 4	15.63	0.45	16.92	0.62	1.29	−0.22 - 2.80	0.14	0.64
**SNAP Hyp/Imp**								
Baseline	16.08	0.56	14.36	0.79				
Week 1	14.54	0.56	13.08	0.79	1.46	−0.44 - 3.36	0.12	
Week 2	13.37	0.56	12.20	0.79	1.16	−0.74 - 3.07	0.10	
Week 3	12.51	0.56	11.13	0.79	1.38	−0.53 - 3.28	0.12	
Week 4	12.32	0.56	11.59	0.79	0.73	−1.17 - 2.63	0.06	0.50
**Conners Teacher - IN**	**Active (n=106)**		**Control (n=55)**					
Baseline	57.71	1.03	59.72	1.44				
Week 4	57.31	1.04	59.05	1.46	1.73	−1.81 - 5.27	0.09	0.82
**Conners Teacher - Hyp**								
Baseline	57.89	1.13	57.14	1.57				
Week 4	57.06	1.14	57.71	1.61	0.83	−0.85 - 2.51	0.09	0.34
**Conners Teacher - Imp**								
Baseline	55.78	1.11	55.94	1.53				
Week 4	56.32	1.12	56.75	1.57	0.54	−1.23 - 2.32	0.13	0.86

Note. ADHDRS: ADHD Rating Scale – clinician rated; SNAP: Swanson, Nolan and Pelham -parent-rated; Conners Teacher Rating Scale – teacher rated. aMD ; average Mean Difference; CI: Confidence Interval; Cohen’s *d* was calculated using the pooled baseline standard deviation of each efficacy measure.

**Table ED3.** ADHD symptom scores by Responder Status

	Responders (n=59)		Nonresponders (n=152)					
	Mean	Std. Error	Mean	Std Error	aMD	95% CI	Cohen’s *d*	group x time p
**ADHDRS Total**								
Baseline	34.76	1.05	35.93	0.65				
Week 1	27.46	1.06	31.22	0.66	3.76	1.35 - 6.18	0.29	
Week 2	23.53	1.05	30.26	0.66	6.73	4.32 - 9.14	0.52	
Week 3	19.70	1.05	29.29	0.66	9.59	7.18 - 12.01	0.74	
Week 4	17.96	1.04	30.59	0.66	12.64	10.25 - 15.03	0.97	<0.001
**ADHDRS Inattention**								
Baseline	19.11	0.55	20.03	0.34				
Week 1	15.58	0.55	17.39	0.34	1.81	0.55 - 3.08	0.26	
Week 2	13.23	0.55	16.85	0.34	3.62	2.36 - 4.88	0.52	
Week 3	11.06	0.55	16.33	0.34	5.27	4.00 - 6.53	0.76	
Week 4	10.15	0.54	17.12	0.34	6.97	5.71 - 8.22	1.00	<0.001
**ADHDRS Hyp/Imp**								
Baseline	15.52	0.76	15.95	0.48				
Week 1	11.76	0.76	13.88	0.48	2.12	0.40 - 3.83	0.22	
Week 2	10.18	0.76	13.46	0.48	3.27	1.56 - 4.99	0.35	
Week 3	8.52	0.76	13.01	0.48	4.49	2.77 - 6.21	0.47	
Week 4	7.69	0.75	13.53	0.48	5.84	4.14 - 7.54	0.62	<0.001
**SNAP Total**								
Baseline	35.33	1.18	36.12	0.73				
Week 1	29.23	1.19	33.30	0.74	4.08	1.36 - 6.79	0.27	
Week 2	25.02	1.18	30.91	0.74	5.89	3.18 - 8.60	0.39	
Week 3	20.27	1.18	30.27	0.74	10.00	7.29 – 12.72	0.66	
Week 4	20.74	1.17	31.07	0.74	10.33	7.64 - 13.01	0.68	<0.001
**SNAP Inattention**								
Baseline	20.44	0.65	20.40	0.40				
Week 1	16.65	0.65	18.69	0.40	2.03	0.54 - 3.53	0.25	
Week 2	14.08	0.65	17.15	0.40	3.07	1.58 - 4.56	0.37	
Week 3	11.38	0.65	17.01	0.41	5.63	4.13 - 7.12	0.68	
Week 4	12.08	0.64	17.65	0.41	5.57	4.09 - 7.04	0.67	<0.001
**SNAP Hyp/Imp**								
Baseline	14.68	0.82	15.80	0.51				
Week 1	12.37	0.82	14.69	0.52	2.32	0.47 - 4.17	0.23	
Week 2	10.74	0.81	13.83	0.52	3.08	1.24 - 4.93	0.30	
Week 3	8.69	0.81	13.35	0.52	4.66	2.81 - 6.51	0.46	
Week 4	8.46	0.81	13.51	0.52	5.04	3.22 - 6.87	0.50	<0.001
**Conners Teacher - IN**	**Responders (n=34)**		**Nonresponders (n=95)**					
Baseline	58.44	1.63	58.39	0.95				
Week 4	56.32	1.59	58.49	0.98	2.17	−1.43 - 5.76	0.16	0.095
**Conners Teacher - HYP**								
Baseline	58.03	1.80	57.51	1.05				
Week 4	55.39	1.75	57.97	1.08	2.59	−1.40 - 6.57	0.16	0.05
**Conners Teacher - IMP**								
Baseline	55.95	1.77	55.80	1.02				
Week 4	54.54	1.72	57.19	1.06	2.65	−1.28 - 6.57	0.16	0.093

Note. Responder: Clinical Global Impression of improvement (CGII) score of 1 (very much improved) or 2 (much improved); Nonresponder = CGII score of 3 or higher. ADHDRS: ADHD Rating Scale – clinician rated; SNAP: Swanson, Nolan and Pelham -parent-rated; Conners Teacher Rating Scale – teacher rated. aMD ; average Mean Difference; CI: Confidence Interval; Cohen’s *d* was calculated using the pooled baseline standard deviation of each efficacy measure.

**Table ED4.** Frontal EEG power by treatment condition/responder status and biological sex

	Treatment condition (n=196)			Responder Status (n=196)		
	*F 1,194 (group)*	*F 1,194 (time)*	*F 1,194 (group x time)*	*F 1,194(group)*	*F 1,194 (time)*	*F 1,194(group x time)*
**Total Sample**						
F4 theta	0.01	0.09	0.94	0.29	2.33	4.65*
F4 alpha	0.01	3.97*	5.66*	1.96	**10.2** ***	1.59
FC4 theta	0.47	0.61	0.19	0.06	2.12	4.10*
FC4 alpha	1.05	3.07^	0.59	0.18	**7.36** **	3.23^
						
Fz theta	0.09	1.14	1.42	0.66	0.10	5.04*
Fz alpha	0.02	2.11	4.13*	2.07	7.6**	2.77^
FCz theta	2.36	0.51	0.53	0.09	3.39^	4.56*
FCz alpha	1.68	3.52^	1.7	0.66	**9.14** ***	3.41^
						
F3 theta	0.96	0.01	0.42	0.23	0.07	0.09
F3 alpha	0.23	1.4	1.99	0.20	3.35^	0.33
FC3 theta	0.91	0.24	4.39*	0.71	0.611	1.81
FC3 alpha	0.14	0.41	**7.22** **	0.09	4.53*	2.39
**Boys**	*F 1,141 (group)*	*F 1,141 (time)*	*F 1,141 (group x time)*	*F 1,141 (group)*	*F 1,141 (time)*	*F 1,141 group x time*
F4 theta	0.01	1.12	2.57	0.47	6.34*	5.87*
F4 alpha	0.83	**8.23** ***	**7.99** ***	**19.23** ****	2.64	3.26
FC4 theta	0.39	0.8	2.52	0.06	7.93**	**11.86** ****
FC4 alpha	0.02	4.89*	6.56*	0.09	**15.94** ****	5.72*
						
Fz theta	0.35	0.41	3.96*	0.51	0.57	3.20^
Fz alpha	1.16	2.88^	5.51*	1.03	**8.48** ***	1.33
FCz theta	1.72	0.51	4.16*	0.16	5.27*	5.07*
FCz alpha	0.21	3.4^	5.98*	0.001	**11.35** ****	3.41^
						
F3 theta	0.19	0.002	2.3	0.09	0.68	0.78
F3 alpha	0.25	1.14	3.46^	0.11	4.24*	0.90
FC3 theta	1.01	0.91	**10.53** ****	0.84	3.23^	4.21*
FC3 alpha	0.04	0.87	**11.46** ****	0.08	**7.34** **	2.89^
**Girls**	*F 1,51 (group)*	*F 1,51 (time)*	*F 1,51 (group x time)*	*F 1,51 (group)*	*F 1,51 (time)*	*F 1,51 group x time*
F4 theta	0.17	0.53	0.04	0.33	0.23	0.14
F4 alpha	2.00	0.003	0.24	0.95	0.001	0.004
FC4 theta	0.6	0.02	3.76^	0.55	0.31	0.58
FC4 alpha	3.5^	0.03	2.12	0.86	0.06	0.005
						
Fz theta	0.14	0.91	0.18	0.04	0.07	1.58
Fz alpha	1.91	0.05	0.14	0.61	0.85	1.32
FCz theta	0.56	0.04	0.98	1.58	0.09	0.47
FCz alpha	2.41	0.44	0.57	1.85	0.52	0.35
						
F3 theta	0.85	0.03	0.56	0.002	0.50	0.50
F3 alpha	1.88	0.26	0.001	0.34	0.06	0.12
FC3 theta	0.02	0.39	0.007	0.12	0.47	0.05
FC3 alpha	0.5	0.02	0.07	0.08	0.009	0.07

Note.

^ p≤0.10,

* p≤0.05,

** p<0.01,

*** p<0.005,

**** p<0.001.

All p-values are uncorrected. Theta power: 4-7 Hz; Alpha power 8-12 Hz; F=Frontal; FC=Frontocentral

**Table ED5.** ADHD symptom scores and CGII responder rate by sex assigned at birth: Girls

	Active (n=36)		Control (n=20)					
	Mean	Std. Error	Mean	Std Error	aMD	95% CI	Cohen’s *d*	group x time p
**ADHDRS Total**								
Baseline	36.49	1.50	34.55	1.95				
Week 1	29.95	1.50	29.23	1.95	0.72	−4.17 - 5.61	0.05	
Week 2	27.27	1.51	25.14	1.95	2.14	−2.76 - 7.04	0.14	
Week 3	27.51	1.52	24.23	1.95	3.28	−1.63 - 8.19	0.21	
Week 4	27.73	1.52	25.05	1.95	2.69	−2.22 - 7.60	0.17	0.671
**ADHDRS Inattention**								
Baseline	20.46	0.76	20.09	0.99				
Week 1	16.57	0.76	17.50	0.99	0.93	−1.54 - 3.40	0.12	
Week 2	15.03	0.77	15.23	0.99	0.20	−2.28 - 2.68	0.03	
Week 3	15.61	0.77	14.68	0.99	0.92	−1.56 - 3.41	0.12	
Week 4	15.92	0.77	14.96	0.99	0.97	−1.52 - 3.45	0.12	0.431
**ADHDRS Hyp/Imp**								
Baseline	16.03	1.03	14.46	1.33				
Week 1	13.38	1.03	11.73	1.33	1.65	−1.70 - 5.01	0.16	
Week 2	12.25	1.03	9.91	1.33	2.34	−1.02 - 5.69	0.22	
Week 3	11.91	1.03	9.55	1.33	2.36	−0.10 - 5.72	0.22	
Week 4	11.82	1.03	10.09	1.33	1.73	−1.63 - 5.09	0.16	0.816
**SNAP Total**								
Baseline	36.43	1.60	34.59	2.07				
Week 1	34.58	1.62	31.47	2.09	3.11	−2.13 - 8.36	0.19	
Week 2	28.48	1.62	26.73	2.07	1.75	−3.47 - 6.97	0.11	
Week 3	27.41	1.63	24.85	2.09	2.56	−2.70 - 7.82	0.16	
Week 4	29.22	1.62	25.86	2.07	3.35	−1.87 - 8.57	0.20	0.907
**SNAP Inattention**								
Baseline	20.87	0.85	21.00	1.10				
Week 1	19.82	0.86	19.43	1.11	0.40	−2.38 - 3.18	0.04	
Week 2	16.13	0.86	15.91	1.10	0.22	−2.53 - 2.98	0.03	
Week 3	15.76	0.87	15.14	1.11	0.62	−2.17 - 3.41	0.07	
Week 4	16.92	0.86	15.91	1.10	1.01	−1.75 - 3.77	0.11	0.93
**SNAP Hyp/Imp**								
Baseline	15.57	1.11	13.59	1.44				
Week 1	14.75	1.11	12.06	1.44	2.69	−0.95 - 6.32	0.24	
Week 2	12.34	1.12	10.82	1.44	1.52	−2.11 - 5.14	0.13	
Week 3	11.66	1.12	9.73	1.44	1.93	−1.71 - 5.57	0.17	
Week 4	12.32	1.12	9.96	1.44	2.37	−1.26 - 5.99	0.21	0.836
					OR	95% CI		
**CGII response rate**	26.50%		15%		2.19	0.52 - 9.20		0.328

Note. ADHDRS: ADHD Rating Scale – clinician rated; SNAP: Swanson, Nolan and Pelham - parent-rated; CGII: Clinical Global Impression of improvement, response = 1 (very much improved) or 2 (much improved); nonresponder = CGI score of 3+, aMD ; average Mean Difference; CI: Confidence Interval; Cohen’s *d* was calculated using the pooled baseline standard deviation of each efficacy measure.

**Table ED6.** ADHD symptom scores and CGII responder rate by sex assigned at birth: Boys

	Active (n=105)		Control (n=50)					
	Mean	Std. Error	Mean	Std Error	aMD	95% CI	Cohen’s *d*	group x time p
**ADHDRS Total**								
Baseline	35.62	0.86	35.41	1.26				
Week 1	30.11	0.87	30.90	1.26	0.79	−2.22 - 3.80	0.05	
Week 2	29.06	0.87	29.28	1.26	0.22	−2.79 - 3.22	0.01	
Week 3	26.65	0.87	27.01	1.26	0.37	−2.65 - 3.39	0.02	
Week 4	26.38	0.87	28.67	1.26	2.29	−0.72 - 5.30	0.15	0.296
**ADHDRS Inattention**								
Baseline	19.23	0.45	20.31	0.66				
Week 1	16.37	0.46	17.94	0.66	1.57	−0.01 - 3.15	0.20	
Week 2	15.82	0.46	16.78	0.66	0.96	−0.62 - 2.54	0.12	
Week 3	14.27	0.46	15.67	0.66	1.40	−0.18 - 2.99	0.17	
Week 4	14.30	0.46	16.43	0.66	2.13	0.55 - 3.71	0.26	0.491
**ADHDRS Hyp/ Imp**								
Baseline	16.39	0.61	15.10	0.89				
Week 1	13.74	0.61	12.96	0.89	0.78	−1.34 - 2.90	0.07	
Week 2	13.24	0.61	12.49	0.89	0.75	−1.37 - 2.87	0.07	
Week 3	12.38	0.61	11.33	0.89	1.05	−1.08 - 3.17	0.10	
Week 4	12.08	0.61	12.24	0.89	0.15	−1.97 - 2.28	0.01	0.298
**SNAP Total**								
Baseline	36.38	0.95	35.12	1.38				
Week 1	31.66	0.95	31.92	1.38	0.27	−3.03 - 3.56	0.02	
Week 2	29.62	0.95	30.21	1.38	0.59	−2.72 - 3.90	0.03	
Week 3	27.90	0.96	27.68	1.38	0.23	−3.08 - 3.54	0.01	
Week 4	27.50	0.96	29.65	1.38	2.15	−1.15 - 5.45	0.13	0.138
**SNAP Inattention**								
Baseline	20.14	0.52	20.43	0.75				
Week 1	17.17	0.52	18.39	0.75	1.22	−0.58 - 3.02	0.14	
Week 2	15.90	0.52	17.39	0.76	1.49	−0.32 - 3.30	0.17	
Week 3	15.12	0.52	15.94	0.76	0.83	−0.98 - 2.64	0.09	
Week 4	15.18	0.52	17.35	0.75	2.17	0.37 - 3.97	0.24	0.200
**SNAP Hyp/Imp**								
Baseline	16.25	0.65	14.69	0.94				
Week 1	14.48	0.65	13.53	0.94	0.95	−1.30 - 3.20	0.08	
Week 2	13.71	0.65	12.80	0.94	0.91	−1.35 - 3.16	0.08	
Week 3	12.79	0.65	11.74	0.94	1.05	−1.21 - 3.31	0.09	
Week 4	12.32	0.65	12.29	0.94	0.02	−2.23 - 2.28	0.00	0.267
					OR	95% CI		
**CGII response rate**	38%		16%		3.28	1.40 - 7.69		0.006

Note. ADHDRS: ADHD Rating Scale – clinician rated; SNAP: Swanson, Nolan and Pelham - parent-rated; CGII: Clinical Global Impression of improvement, response = 1 (very much improved) or 2 (much improved); nonresponder = CGI score of 3+, aMD ; average Mean Difference; CI: Confidence Interval; Cohen’s *d* was calculated using the pooled baseline standard deviation of each efficacy measure.

**Table ED7.** Treatment emergent side effects: parent report on Side Effects Rating Scale

	Active	Sham
Stuffy nose	12%	18%
Night Wake	12%	10%
Drowsy	10%	15%
Forehead pain	10%	4%
Routine	10%	11%
interference		
Sleep Trouble	9%	12%
Headache	8%	15%
Stomachache	8%	7%
Fatigue	8%	7%
Excessive sweating	1%	6%
Nausea Vomiting	3%	6%

Note. Treatment-emergent side effects captured by the Side Effect Rating Scale (SERS)—restricted to symptoms absent at baseline and reported cumulatively post-randomization. Side effects reported for greater than 5% of sample included here.

**Table ED8.** Treatment emergent adverse events reported

	Device		P-value
	Active	Control	
	n, %	n, %	
No Adverse Events	78 (53.8%)	36 (49.3%)	0.53
Upper_Respiratory_Infection	21 (14.5%)	9 (12.3%)	0.84
Headache	17 (11.7%)	5 (6.8%)	0.34
Nausea	7 (4.8%)	5 (6.8%)	0.54
Application Site Rash	7 (4.8%)	5 (6.8%)	0.43
Fever	5 (3.4%)	0 (0.0%)	0.17
Body_Aches	3 (2.1%)	1 (1.4%)	1.00
Seasonal_allergies	3 (2.1%)	2 (2.7%)	1.00
Influenza	3 (2.1%)	1 (1.4%)	1.00
Contusion	3 (2.1%)	3 (4.1%)	0.40
Abdominal Pain	2 (1.4%)	2 (2.7%)	0.60
Laceration	2 (1.4%)	1 (1.4%)	1.00
Increased_Appetite	2 (1.4%)	0 (0.0%)	0.55
Sleep_disorder	2 (1.4%)	0 (0.0%)	0.55
Asthma	2 (1.4%)	0 (0.0%)	0.55
Constipation	1 (0.7%)	0 (0.0%)	1.00
Tooth_Pain	1 (0.7%)	0 (0.0%)	1.00
Bee Sting	1 (0.7%)	0 (0.0%)	1.00
COVID 19	1 (0.7%)	1 (1.4%)	1.00
Localised_infection	1 (0.7%)	1 (1.4%)	1.00
Decreased Appetite	1 (0.7%)	0 (0.0%)	1.00
Teeth_Grinding	1 (0.7%)	0 (0.0%)	1.00
Nosebleed	1 (0.7%)	1 (1.4%)	1.00
Sweaty_Hands	1 (0.7%)	0 (0.0%)	1.00
Diarrhea	0 (0.0%)	1 (1.4%)	0.33
Chills	0 (0.0%)	1 (1.4%)	0.33
Fatigue	0 (0.0%)	1 (1.4%)	0.33
Enuresis	0 (0.0%)	1 (1.4%)	0.33
Motor_Tic	0 (0.0%)	3 (4.1%)	0.04

## Figures and Tables

**Figure 1.**
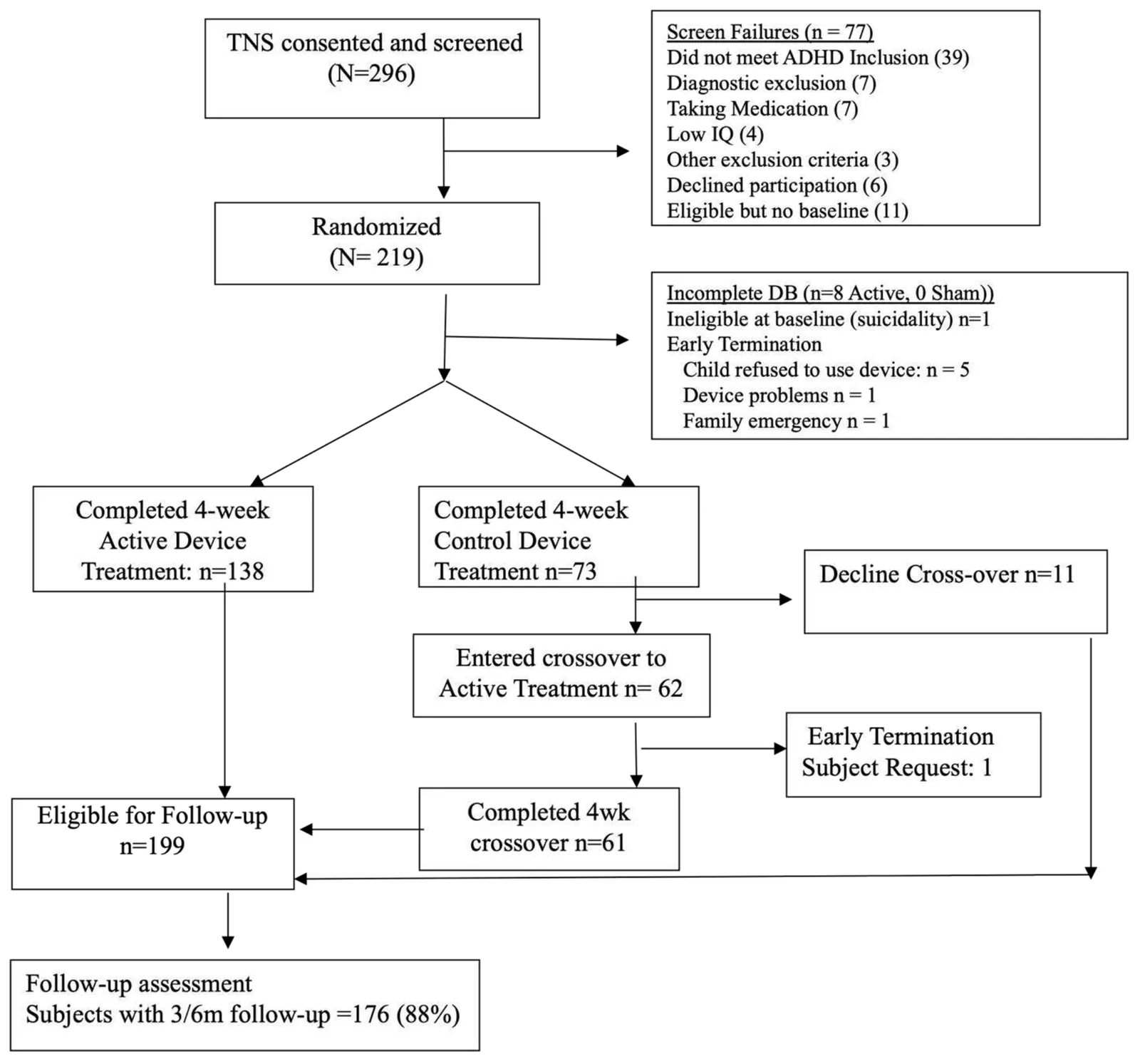
Consort Diagram

**Figure 2.**
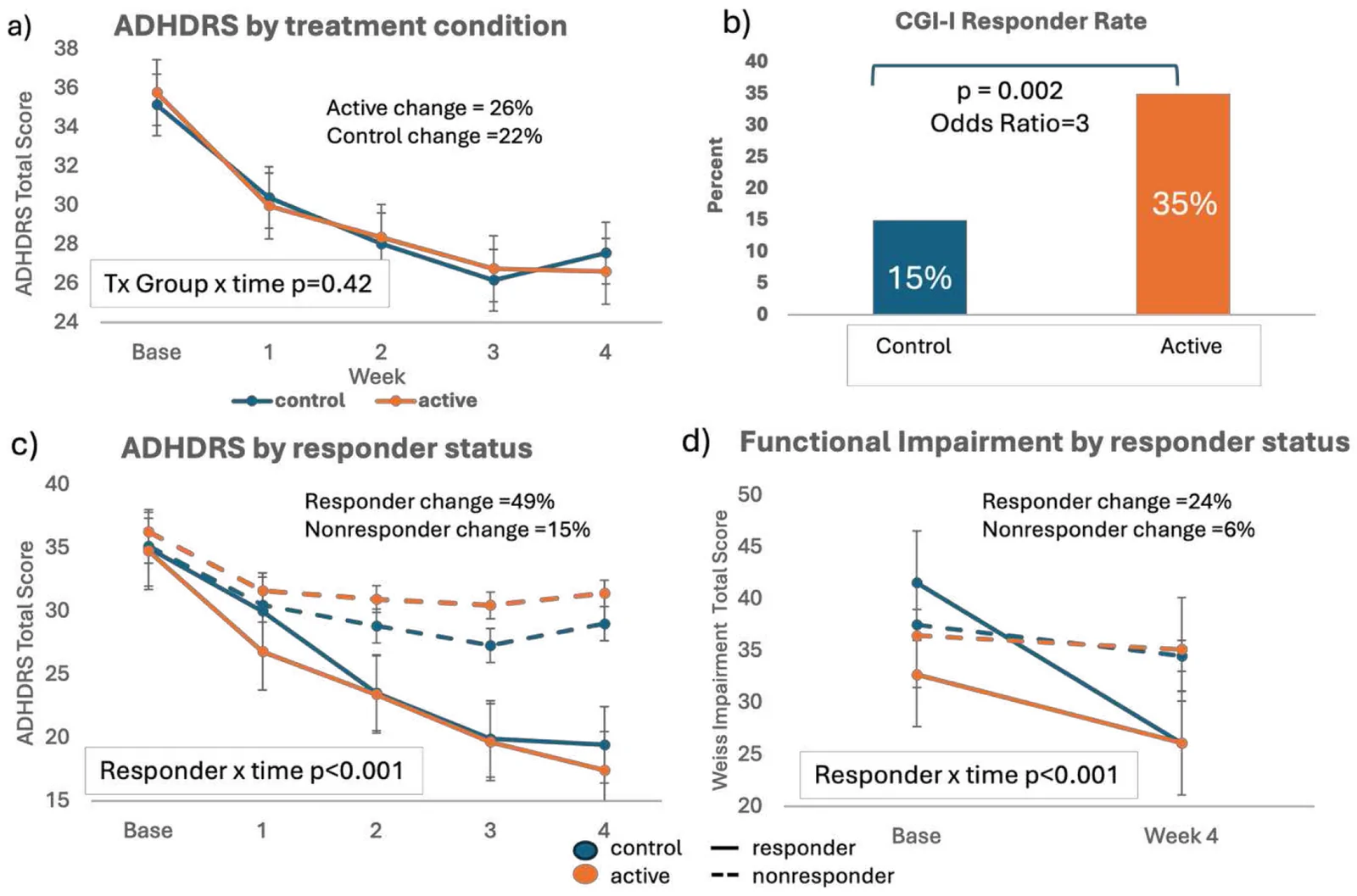
ADHD symptoms and impairment by treatment condition and responder status. Note. a) Both active and control condition showed similar amount of change during four weeks of TNS treatment. b) The active group was three times more likely to be a Clinical Global Impression of Improvement (CGI-I) responder (e.g., CGI-I scores 1 [very much improved] or 2 [much improved] vs nonresponders CGI-I scores [3 or higher] compared to control group. c) CGI-I responders showed significantly more improvement on ADHDRS Total score compared to CGII nonresponders. CGI-I responders had improved Weiss Functional Impairment Rating Scale scores compared to nonresponders.

**Figure 3.**
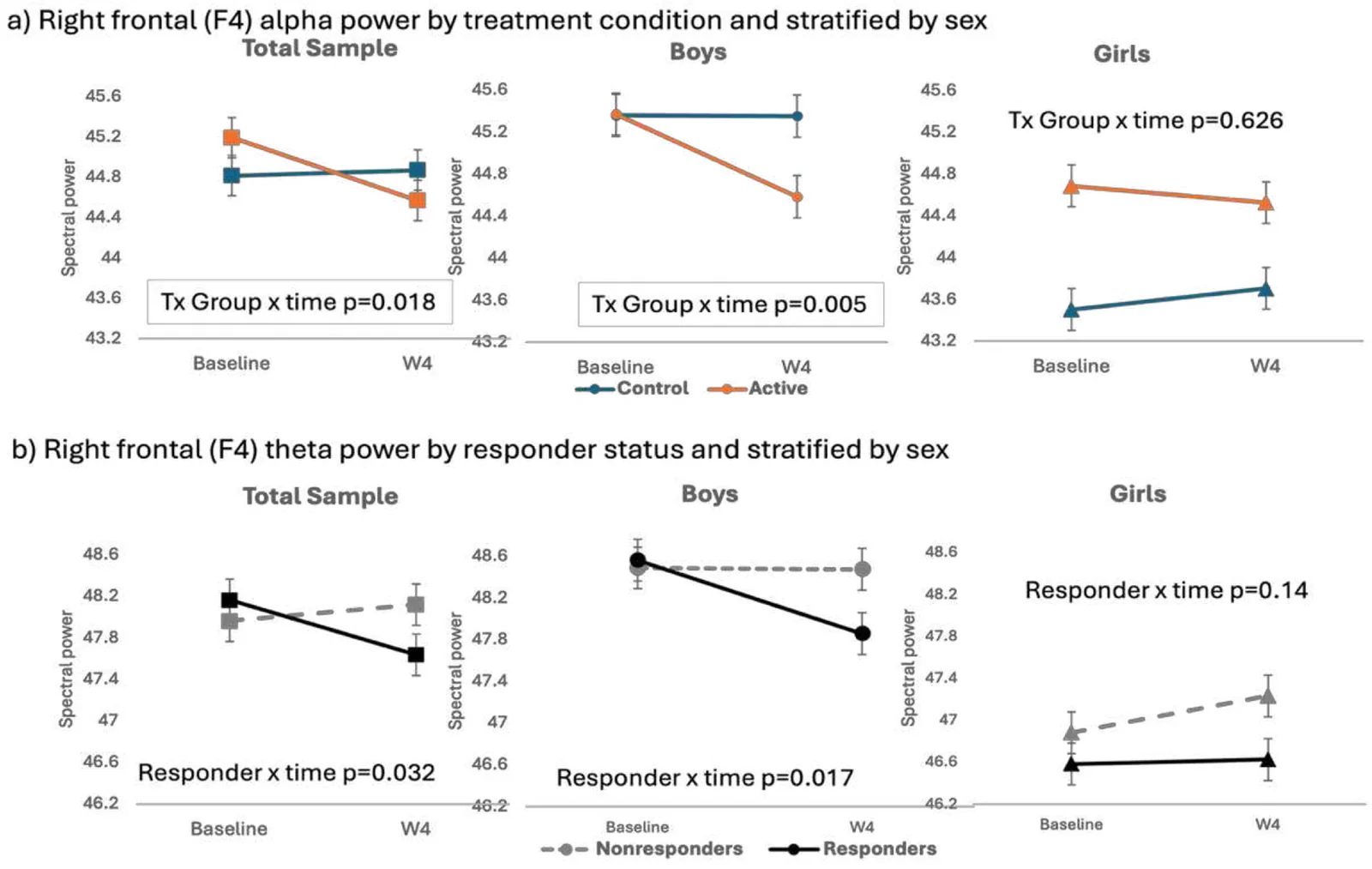
Right frontal EEG power by treatment condition and responder status. Note. Panel a) F4 alpha (8-12 Hz) power treatment group by time interaction p-value in the total sample and stratified by sex assigned at birth. Panel b) F4 theta (4-7 Hz) power responder x time interaction p-value in total sample and stratified by sex.

**Figure 4.**
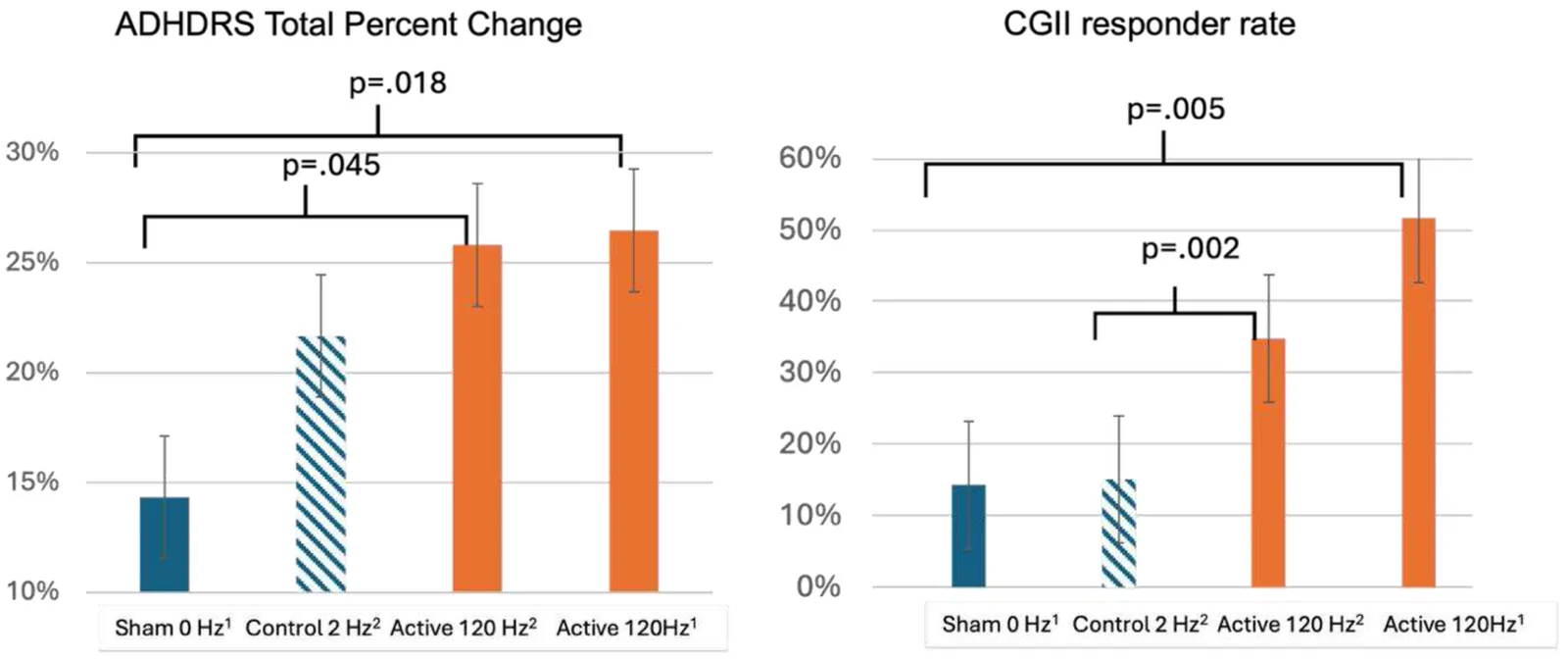
Effects of different stimulation frequencies across studies. *Note*. Cross study comparison of none, low and high frequency TNS treatment. Methodologies for both studies were identical with exception of comparison conditions. ^1^McGough et al., 2019 (N=61); ^2^current study (N=218). Panel a) Pairwise comparisons indicate that both active conditions are significantly different than no stimulation sham condition. The 2Hz control is not different from any of the groups, likely due to low power. Panel b) Responder rate is the same in 0 Hz and 2 Hz conditions, which are both significantly different from active 120 Hz stimulation.

**Table 1.** Participant Baseline Demographics and Clinical Characteristics

	Total Sample (N=218)	Active (n = 145)	Control (n = 73)	Statistic	*p*-value
**Demographic Characteristics**					
Age, years, mean (SD)	9.8 (1.7)	9.9 (1.7)	9.7 (1.8)	t = 0.40	.688
Male, n (%)	159 (73.0)	108 (75.0)	51 (70.0)	χ^2^ = 0.53	.469
Race/Ethnicity, n (%)				χ^2^ = 6.39	.270
White	142 (65%)	89 (61 %)	53 (73%)		
Non-Hispanic/Latino	73 (33.0)	46 (32.0)	27 (37.0)		
Hispanic/Latino	69 (32.0)	43 (30.0)	26 (36.0)		
Black	10 (5.0)	10 (7.0)	0 (0.0)		
Asian	34 (16.0)	24 (17.0)	10 (14.0)		
Mixed Race	28 (13.0)	19 (13.0)	9 (12.0)		
Other	4 (2.0)	3 (2.1)	1 (1.4)		
Hollingshead SES rank	2.02 (.9)	2.1 (.9)	1.9 (.9)	t = 1.13	.290
**Clinical Characteristics**					
Height, cm, mean (SD)	139.0 (11.9)	139.1 (11.6)	138.8 (12.7)	t = 0.16	.876
Weight, kg, mean (SD)	36.0 (11.4)	36.4 (11.5)	35.3 (11.3)	t = 0.69	.494
Full-Scale IQ, mean (SD)	110.6 (13.8)	111.5 (14.2)	108.8 (13.1)	t = 1.36	.176
ADHD Presentation, n (%)				χ^2^ = 2.79	.247
Combined	121 (55.5)	82 (56.6)	39 (53.4)		
Hyperactive/Impulsive	13 (6.0)	11 (7.6)	2 (2.7)		
Inattentive	84 (38.5)	52 (35.9)	32 (43.8)		
ADHD-RS Total Score, mean (SD)	35.6 (7.0)	35.8 (7.1)	35.2 (6.9)	t = 1.04	.300
CGI-S Score, mean (SD)	4.5 (0.5)	4.5 (0.5)	4.4 (0.5)	t = 1.39	.165
**Comorbid Diagnoses, n (%)**					
Oppositional Defiant Disorder (ODD)	49 (22.5)	33 (22.8)	16 (21.9)	χ^2^ = 0.03	.857
Disruptive Mood Dysregulation	3 (1.4)	1 (0.7)	2 (2.7)	Fisher’s Exact	.220
Disorder (DMDD)					
Obsessive-Compulsive Disorder	1 (0.5)	1 (0.7)	0 (0.0)	Fisher’s Exact	.477
Any Anxiety Disorder	60 (27.5)	35 (24.1)	25 (34.2)	χ^2^ = 2.49	.115
panic Disorder	1 (0.5)	1 (0.7)	0 (0.0)		
Separation Anxiety	3(1.4)	2 (1.4)	1 (1.4)		
Social Anxiety	6(2.8)	2 (1.4)	4 (5.5)		
Specific Phobia	19(8.7)	15 (10.3)	4 (5.5)		
Generalized Anxiety Disorder	36(16.5)	18 (12.4)	18 (24.7)		
Enuresis/Encopresis	10 (4.6)	5 (3.4)	5 (6.8)	Fisher’s Exact	.257
Chronic Tic Disorder	18 (8.3)	11 (7.6)	7 (9.6)	χ^2^ = 0.26	.612

Note. Race/ethnicity: White racial group is split to reflect two ethnicities; ‘Other’ category includes Hawaiian/Pacific Islander and Native American. Hollingshead^54^ socioeconomic status (SES) rank range is 1(highest) to 5 (lowest). CGI-S: Clinical Global Impression-Severity^14^. ADHD and other diagnoses were determined using the Kiddie Schedule for Affective Disorders and Schizophrenia for School-Age Children^41^ (KSADS).

**Table 2.** Pearson Correlations between TNS Stimulation Metrics and EEG Power

	Active		Control	
	Hours per week	Intensity	Hours per week	Intensity
F4 theta	0.11	−0.02	0.15	**0.34** **
F4 alpha	0.12	0.1	0.02	0.23
F4 beta	0.07	0.04	0.16	**0.25** *
Fz theta	−0.04	0.12	**0.42** ****	0.21
Fz alpha	−0.02	0.17	**0.28** *	0.12
Fz beta	−0.03	0.17	**0.39** ***	0.15
FC4 theta	0.05	0.01	0.11	**0.43** ****
FC4 alpha	0.03	0.12	−0.04	**0.30** *
FC4 beta	0.08	0.1	−0.05	**0.26** *
FCz theta	0.13	0.15	0.06	**0.30** *
FCz alpha	0.13	**0.19** *	−0.02	0.24
FCz beta	0.11	**0.21** *	0.09	**0.30** *

Note.

* p< 0.05,

** p<0.01,

*** p<0.005,

**** p< 0.001.

Pearson two-tailed correlations between parent-reported total hours per week that device was worn (Hrs per week) and stimulation level (mA; intensity) and EEG spectral power in theta (4-7 Hz), alpha (8-12 Hz), and beta (13-25 Hz) bands shown separately by treatment condition. F: frontal & F:-Fronto-central electrode location.

## Data Availability

De-identified individual participant data and associated data dictionaries have been deposited into the NIH National Institute of Mental Health Data Archive (NDA) collection 4315.

## Code availability:

No custom or unpublished software was used.
